# Trichlorido-1κ^2^ *Cl*,2κ*Cl*-(2,6-dimethyl­phenolato-2κ*O*)-μ-oxido-bis{1,2(η^5^)-2,3,4,5-tetra­methyl-1-[4-(trimethyl­silyl)phen­yl]cyclo­penta­dien­yl}dititanium(IV)

**DOI:** 10.1107/S1600536811035306

**Published:** 2011-09-14

**Authors:** Xuyang Luo, Qiaolin Wu, Ying Mu

**Affiliations:** a School of Chemistry, Jilin University, Changchun 130012, People’s Republic of China; b State Key Laboratory of Supramolecular Structure and Materials, School of Chemistry, Jilin University, Changchun 130012, People’s Republic of China

## Abstract

The title dinuclear titanocene, [Ti_2_(C_8_H_9_O)(C_18_H_25_Si)_2_Cl_3_O], contains one Ti atom tetra­hedrally coordinated by two Cl atoms, a bridging O atom and the substituted cyclo­penta­dienyl ligand, and another Ti atom tetra­hedrally coordinated by a Cl atom, a bridging O atom, the 2,6-dimethyl­phenolate ligand and the substituted cyclo­penta­dienyl ligand. The bridging O atom lies on a twofold rotation axis.

## Related literature

For background to titanocene complexes, see: Bochmann (2010 ▶); Lee *et al.* (2001 ▶); Wu *et al.* (2006 ▶). For potential applications in olefin polymerization, see: Blais *et al.* (1998 ▶); Wilson *et al.* (2008 ▶). For Ti—O—Ti angles in related structures, see: Ciruelous *et al.* (1993 ▶); Varkey *et al.* (2001 ▶). For the preparation, see: Wu *et al.* (2007 ▶, 2010 ▶).
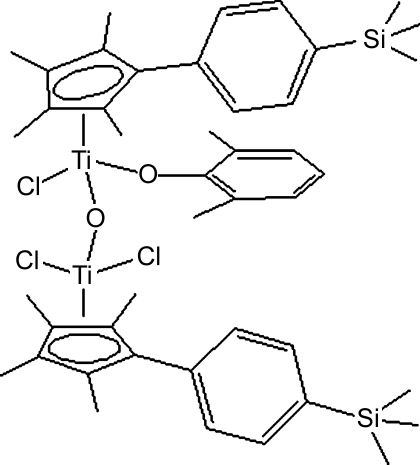


## Experimental

### 

#### Crystal data

[Ti_2_(C_8_H_9_O)(C_18_H_25_Si)_2_Cl_3_O]
*M* *_r_* = 878.24
Triclinic, 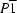

*a* = 11.405 (2) Å
*b* = 12.949 (3) Å
*c* = 18.132 (4) Å
α = 104.19 (3)°
β = 101.13 (3)°
γ = 108.96 (3)°
*V* = 2344.2 (8) Å^3^
*Z* = 2
Mo *K*α radiation
μ = 0.60 mm^−1^
*T* = 293 K
0.21 × 0.18 × 0.13 mm

#### Data collection

Bruker P4 diffractometer
Absorption correction: multi-scan (*SADABS*; Bruker, 2001 ▶) *T*_min_ = 0.882, *T*_max_ = 0.925
22084 measured reflections
10372 independent reflections
6422 reflections with *I* > 2σ(*I*)
*R*_int_ = 0.039

#### Refinement

*R*[*F* ^2^ > 2σ(*F* ^2^)] = 0.057
*wR*(*F* ^2^) = 0.188
*S* = 1.05
10372 reflections
494 parameters
6 restraints
H-atom parameters constrained
Δρ_max_ = 0.35 e Å^−3^
Δρ_min_ = −0.39 e Å^−3^

### 

Data collection: *XSCANS* (Bruker, 1998 ▶); cell refinement: *XSCANS*; data reduction: *XSCANS*; program(s) used to solve structure: *SHELXS97* (Sheldrick, 2008 ▶); program(s) used to refine structure: *SHELXL97* (Sheldrick, 2008 ▶); molecular graphics: *SHELXTL* (Sheldrick, 2008 ▶); software used to prepare material for publication: *SHELXTL*.

## Supplementary Material

Crystal structure: contains datablock(s) global. DOI: 10.1107/S1600536811035306/qm2025sup1.cif

Additional supplementary materials:  crystallographic information; 3D view; checkCIF report

## Figures and Tables

**Fig. 1.**
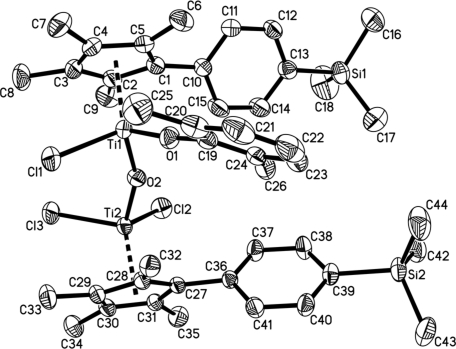
View of the molecule of (I) showing the atom labelling scheme. Displacement ellipsoids are drawn at the 30% probability level. The hydrogen atoms are omitted for clarity.

## supplementary crystallographic information

### Comment

Group 4 metallocene complexes with desirable steric and electronic properties have been of considerable interest in recent years, due to the potential applications in olefin polymerization. The most used strategy for improving catalyst performance is modification of the ligand framework by rational tailoring of steric and electronic factors. (Blais, *et al.*, 1998; Wilson, *et al.*, 2008) In our previous work, we have reported a series of monocyclopentadienyl titanium complexes and some of their hydrolysis products (Wu *et al.*, 2006, 2007, 2010). It is of significance to clarify the struture of the hydrolysis products. Therefore, we report herein the crystal structure of the title compound (I)(shown in Fig. 1).

The title compound shows a bimetallic moiety with two cyclopentadienyltitanium units linked by Ti—O bonding. The bridging O atom lies on a crystallographic twofold axis. The title titanocene features tetrahedral coordination geometry around the titanium atom, formed by a substituted cyclopentadienyl ring, two chloride atoms (a chloride atom and a phenolate O atom) and an oxygen atom (oxo-bridge). The average C—C distance of Cp rings (1.417 (5) and 1.423 (4) Å for C1—C5 and C27—C31, respectively) is somewhat longer than the average values in substituted phenyl groups (1.387 (5) Å for both C10—C15 and C36—C41). The Ti—O—Ti angle of 157.0 ° falls within the range of observed values (154–180 °) in titanocene analogues, indicative of resulting from different pi back-bonding affected by intramolecular steric effects (Varkey *et al.*, 2001; Ciruelous *et al.*, 1993) The dihedral angles between the Cp rings and the adjacent phenyl rings are 51.3 ° (C1—C5 and C10—C15), 57.2 ° (C27—C31 and C36—C41),respectively, which is related to the steric crowding of substituted Cp ring through steric and electronic effects of aromatic substituents attached to the Cp ring. The two Cp rings are nearly parallel, with a dihedral angle of 4.2 °.

### Experimental

Compound (I) was prepared as described in the litererature (Wu *et al.*, 2007, 2010) with {1-(4-trimethylsilylphenyl)-2,3,4,5-tetramethyl- cyclopentadienyl}titanium(IV) trichloride and 2,6-dimethylphenol as starting material. Crystals suitable for X-ray analysis were obtained by recrystallization from a mixture of dichloromethane and n-hexane (1:5 *v*/*v*) at room temperature.

### Refinement

The C-bound H atoms were positioned geometrically with C—H = 0.93 and 0.96 Å, for aromatic and methyl H-atoms, respectively, and allowed to ride on their parent atoms in the riding model approximation with *U*_iso_(H) = 1.2 *U*_eq_(C) for aromatic H-atoms or 1.5 *U*_eq_(C) for methyl H-atoms.

### Crystal data

**Table:**

[Ti_2_(C_8_H_9_O)(C_18_H_25_Si)_2_Cl_3_O]	*Z* = 2
*M**_r_* = 878.24	*F*(000) = 924
Triclinic, *P*1	*D*_x_ = 1.244 Mg m^−^^3^
Hall symbol: -P 1	Mo *K*α radiation, λ = 0.71073 Å
*a* = 11.405 (2) Å	Cell parameters from 15652 reflections
*b* = 12.949 (3) Å	θ = 3.0–27.4°
*c* = 18.132 (4) Å	µ = 0.60 mm^−^^1^
α = 104.19 (3)°	*T* = 293 K
β = 101.13 (3)°	Block, red
γ = 108.96 (3)°	0.21 × 0.18 × 0.13 mm
*V* = 2344.2 (8) Å^3^	

### Data collection

**Table:**

Bruker P4 diffractometer	10372 independent reflections
Radiation source: fine-focus sealed tube	6422 reflections with *I* > 2σ(*I*)
graphite	*R*_int_ = 0.039
ω scans	θ_max_ = 27.5°, θ_min_ = 3.1°
Absorption correction: multi-scan (*SADABS*; Bruker, 2001)	*h* = −14→14
*T*_min_ = 0.882, *T*_max_ = 0.925	*k* = −15→16
22084 measured reflections	*l* = −23→23

### Refinement

**Table:**

Refinement on *F*^2^	Primary atom site location: structure-invariant direct methods
Least-squares matrix: full	Secondary atom site location: difference Fourier map
*R*[*F*^2^ > 2σ(*F*^2^)] = 0.057	Hydrogen site location: inferred from neighbouring sites
*wR*(*F*^2^) = 0.188	H-atom parameters constrained
*S* = 1.05	*w* = 1/[σ^2^(*F*_o_^2^) + (0.1012*P*)^2^ + 0.0969*P*] where *P* = (*F*_o_^2^ + 2*F*_c_^2^)/3
10372 reflections	(Δ/σ)_max_ = 0.003
494 parameters	Δρ_max_ = 0.35 e Å^−^^3^
6 restraints	Δρ_min_ = −0.39 e Å^−^^3^

### Special details

**Table:**

Experimental. see experiment
Geometry. All e.s.d.'s (except the e.s.d. in the dihedral angle between two l.s. planes) are estimated using the full covariance matrix. The cell e.s.d.'s are taken into account individually in the estimation of e.s.d.'s in distances, angles and torsion angles; correlations between e.s.d.'s in cell parameters are only used when they are defined by crystal symmetry. An approximate (isotropic) treatment of cell e.s.d.'s is used for estimating e.s.d.'s involving l.s. planes.
Refinement. Refinement of *F*^2^ against ALL reflections. The weighted *R*-factor *wR* and goodness of fit *S* are based on *F*^2^, conventional *R*-factors *R* are based on *F*, with *F* set to zero for negative *F*^2^. The threshold expression of *F*^2^ > σ(*F*^2^) is used only for calculating *R*-factors(gt) *etc*. and is not relevant to the choice of reflections for refinement. *R*-factors based on *F*^2^ are statistically about twice as large as those based on *F*, and *R*- factors based on ALL data will be even larger.

### Fractional atomic coordinates and isotropic or equivalent isotropic displacement parameters (Å^2^)

**Table:**

	*x*	*y*	*z*	*U*_iso_*/*U*_eq_	
Ti1	0.05505 (6)	0.33102 (5)	0.23000 (4)	0.04577 (18)	
Ti2	−0.09126 (5)	0.53445 (5)	0.27494 (4)	0.04253 (17)	
Cl1	−0.14294 (10)	0.18775 (9)	0.15420 (7)	0.0745 (3)	
Cl2	0.02690 (10)	0.68549 (9)	0.38951 (6)	0.0637 (3)	
Cl3	−0.23923 (10)	0.42014 (10)	0.31861 (7)	0.0759 (3)	
Si1	0.68227 (11)	0.86972 (10)	0.48153 (7)	0.0659 (3)	
Si2	0.44356 (10)	0.96451 (9)	0.15117 (7)	0.0589 (3)	
O1	0.1325 (2)	0.3496 (2)	0.15419 (15)	0.0550 (6)	
O2	0.0119 (2)	0.45837 (19)	0.25895 (14)	0.0487 (6)	
C1	0.2305 (3)	0.3869 (3)	0.3443 (2)	0.0522 (9)	
C2	0.1156 (4)	0.3543 (4)	0.3693 (2)	0.0618 (11)	
C3	0.0423 (4)	0.2359 (4)	0.3271 (3)	0.0688 (12)	
C4	0.1105 (4)	0.1936 (3)	0.2787 (3)	0.0653 (11)	
C5	0.2272 (4)	0.2861 (3)	0.2893 (2)	0.0558 (9)	
C6	0.3312 (4)	0.2789 (4)	0.2507 (3)	0.0720 (12)	
H6A	0.3963	0.2665	0.2855	0.108*	
H6B	0.3702	0.3498	0.2407	0.108*	
H6C	0.2934	0.2156	0.2012	0.108*	
C7	0.0699 (5)	0.0681 (3)	0.2272 (4)	0.0976 (18)	
H7A	0.0913	0.0251	0.2601	0.146*	
H7B	0.1148	0.0655	0.1876	0.146*	
H7C	−0.0221	0.0348	0.2015	0.146*	
C8	−0.0843 (4)	0.1661 (5)	0.3372 (3)	0.1008 (19)	
H8A	−0.1519	0.1366	0.2876	0.151*	
H8B	−0.1056	0.2144	0.3772	0.151*	
H8C	−0.0762	0.1027	0.3532	0.151*	
C9	0.0840 (5)	0.4291 (4)	0.4330 (3)	0.0777 (13)	
H9A	−0.0020	0.3881	0.4344	0.117*	
H9B	0.0883	0.4987	0.4218	0.117*	
H9C	0.1454	0.4485	0.4837	0.117*	
C10	0.3386 (3)	0.5025 (3)	0.3761 (2)	0.0500 (8)	
C11	0.4654 (4)	0.5133 (3)	0.4047 (2)	0.0556 (9)	
H11	0.4821	0.4473	0.4030	0.067*	
C12	0.5676 (4)	0.6208 (3)	0.4359 (2)	0.0571 (9)	
H12	0.6517	0.6254	0.4546	0.069*	
C13	0.5472 (4)	0.7218 (3)	0.4396 (2)	0.0537 (9)	
C14	0.4199 (4)	0.7100 (3)	0.4123 (3)	0.0656 (11)	
H14	0.4034	0.7764	0.4149	0.079*	
C15	0.3171 (4)	0.6046 (3)	0.3816 (3)	0.0660 (11)	
H15	0.2331	0.6008	0.3644	0.079*	
C16	0.8427 (5)	0.8595 (5)	0.5069 (4)	0.1062 (19)	
H16A	0.8548	0.8386	0.5543	0.159*	
H16B	0.9100	0.9330	0.5160	0.159*	
H16C	0.8463	0.8017	0.4637	0.159*	
C17	0.6756 (6)	0.9428 (5)	0.4067 (4)	0.122 (2)	
H17A	0.6121	0.8893	0.3575	0.183*	
H17B	0.7592	0.9702	0.3982	0.183*	
H17C	0.6520	1.0071	0.4252	0.183*	
C18	0.6593 (8)	0.9524 (6)	0.5714 (4)	0.148 (3)	
H18A	0.5775	0.9598	0.5578	0.223*	
H18B	0.7283	1.0279	0.5937	0.223*	
H18C	0.6602	0.9127	0.6097	0.223*	
C19	0.2000 (3)	0.3610 (3)	0.1005 (2)	0.0496 (8)	
C20	0.1831 (4)	0.2629 (4)	0.0395 (2)	0.0652 (10)	
C21	0.2525 (5)	0.2795 (5)	−0.0159 (3)	0.0837 (14)	
H21	0.2455	0.2156	−0.0562	0.100*	
C22	0.3295 (5)	0.3874 (6)	−0.0114 (3)	0.0886 (16)	
H22	0.3728	0.3968	−0.0494	0.106*	
C23	0.3434 (4)	0.4819 (5)	0.0484 (3)	0.0799 (14)	
H23	0.3970	0.5551	0.0509	0.096*	
C24	0.2793 (3)	0.4716 (4)	0.1061 (2)	0.0577 (10)	
C25	0.0946 (5)	0.1436 (4)	0.0317 (3)	0.0923 (16)	
H25A	0.1290	0.1225	0.0758	0.139*	
H25B	0.0882	0.0894	−0.0172	0.139*	
H25C	0.0100	0.1424	0.0317	0.139*	
C26	0.2967 (4)	0.5758 (3)	0.1735 (3)	0.0739 (12)	
H26A	0.2133	0.5770	0.1745	0.111*	
H26B	0.3485	0.6450	0.1658	0.111*	
H26C	0.3394	0.5716	0.2232	0.111*	
C27	−0.0665 (3)	0.6388 (3)	0.18409 (19)	0.0402 (7)	
C28	−0.1364 (3)	0.5201 (3)	0.13894 (19)	0.0422 (7)	
C29	−0.2573 (3)	0.4833 (3)	0.1562 (2)	0.0491 (8)	
C30	−0.2621 (3)	0.5808 (3)	0.2104 (2)	0.0505 (8)	
C31	−0.1453 (3)	0.6770 (3)	0.2277 (2)	0.0455 (8)	
C32	−0.0959 (4)	0.4467 (3)	0.0796 (2)	0.0544 (9)	
H32A	−0.1357	0.4436	0.0270	0.082*	
H32B	−0.1230	0.3699	0.0826	0.082*	
H32C	−0.0032	0.4797	0.0914	0.082*	
C33	−0.3632 (4)	0.3654 (3)	0.1195 (3)	0.0721 (12)	
H33A	−0.4095	0.3488	0.1569	0.108*	
H33B	−0.3263	0.3092	0.1054	0.108*	
H33C	−0.4218	0.3625	0.0725	0.108*	
C34	−0.3748 (4)	0.5838 (4)	0.2402 (3)	0.0738 (12)	
H34A	−0.3436	0.6385	0.2931	0.111*	
H34B	−0.4238	0.5083	0.2409	0.111*	
H34C	−0.4293	0.6064	0.2057	0.111*	
C35	−0.1170 (4)	0.7988 (3)	0.2744 (3)	0.0642 (11)	
H35A	−0.1381	0.8384	0.2387	0.096*	
H35B	−0.0265	0.8373	0.3039	0.096*	
H35C	−0.1681	0.7994	0.3107	0.096*	
C36	0.0589 (3)	0.7159 (3)	0.17851 (19)	0.0399 (7)	
C37	0.1668 (3)	0.7828 (3)	0.2441 (2)	0.0536 (9)	
H37	0.1639	0.7793	0.2944	0.064*	
C38	0.2798 (4)	0.8555 (3)	0.2350 (2)	0.0582 (10)	
H38	0.3518	0.8981	0.2796	0.070*	
C39	0.2891 (3)	0.8669 (3)	0.1625 (2)	0.0487 (8)	
C40	0.1799 (4)	0.7992 (3)	0.0971 (2)	0.0566 (9)	
H40	0.1826	0.8033	0.0469	0.068*	
C41	0.0663 (3)	0.7251 (3)	0.1051 (2)	0.0543 (9)	
H41	−0.0053	0.6813	0.0604	0.065*	
C42	0.5400 (4)	1.0825 (4)	0.2478 (3)	0.0798 (13)	
H42A	0.5715	1.0506	0.2860	0.120*	
H42B	0.4860	1.1196	0.2668	0.120*	
H42C	0.6122	1.1383	0.2403	0.120*	
C43	0.3993 (5)	1.0289 (4)	0.0737 (3)	0.0911 (16)	
H43A	0.3649	1.0845	0.0943	0.137*	
H43B	0.3351	0.9688	0.0272	0.137*	
H43C	0.4752	1.0664	0.0597	0.137*	
C44	0.5408 (5)	0.8797 (4)	0.1237 (4)	0.108 (2)	
H44A	0.6179	0.9285	0.1159	0.162*	
H44B	0.4905	0.8162	0.0753	0.162*	
H44C	0.5646	0.8505	0.1656	0.162*	

### Atomic displacement parameters (Å^2^)

**Table:**

	*U*^11^	*U*^22^	*U*^33^	*U*^12^	*U*^13^	*U*^23^
Ti1	0.0383 (3)	0.0446 (3)	0.0570 (4)	0.0132 (3)	0.0155 (3)	0.0244 (3)
Ti2	0.0357 (3)	0.0491 (3)	0.0466 (4)	0.0152 (3)	0.0168 (3)	0.0206 (3)
Cl1	0.0512 (6)	0.0616 (6)	0.0900 (8)	0.0020 (4)	0.0043 (5)	0.0309 (6)
Cl2	0.0685 (6)	0.0677 (6)	0.0489 (5)	0.0243 (5)	0.0161 (5)	0.0136 (5)
Cl3	0.0552 (6)	0.0938 (8)	0.0904 (8)	0.0178 (5)	0.0329 (6)	0.0567 (7)
Si1	0.0527 (7)	0.0616 (7)	0.0650 (7)	0.0095 (5)	0.0133 (5)	0.0106 (6)
Si2	0.0497 (6)	0.0515 (6)	0.0778 (8)	0.0126 (5)	0.0348 (6)	0.0231 (5)
O1	0.0539 (15)	0.0548 (14)	0.0638 (16)	0.0224 (12)	0.0264 (13)	0.0237 (12)
O2	0.0444 (13)	0.0535 (13)	0.0524 (14)	0.0217 (11)	0.0154 (11)	0.0205 (11)
C1	0.0423 (19)	0.062 (2)	0.058 (2)	0.0214 (16)	0.0140 (17)	0.0290 (19)
C2	0.046 (2)	0.086 (3)	0.067 (3)	0.026 (2)	0.0182 (19)	0.049 (2)
C3	0.048 (2)	0.080 (3)	0.089 (3)	0.016 (2)	0.018 (2)	0.062 (3)
C4	0.055 (2)	0.055 (2)	0.091 (3)	0.0183 (18)	0.011 (2)	0.045 (2)
C5	0.048 (2)	0.052 (2)	0.077 (3)	0.0243 (16)	0.0165 (19)	0.032 (2)
C6	0.057 (3)	0.072 (3)	0.096 (3)	0.035 (2)	0.024 (2)	0.028 (2)
C7	0.089 (4)	0.051 (2)	0.141 (5)	0.017 (2)	0.012 (3)	0.044 (3)
C8	0.058 (3)	0.124 (4)	0.121 (4)	0.004 (3)	0.022 (3)	0.087 (4)
C9	0.074 (3)	0.120 (4)	0.061 (3)	0.047 (3)	0.034 (2)	0.044 (3)
C10	0.0416 (19)	0.056 (2)	0.055 (2)	0.0194 (16)	0.0140 (16)	0.0231 (17)
C11	0.051 (2)	0.058 (2)	0.065 (2)	0.0256 (17)	0.0153 (18)	0.0284 (19)
C12	0.0403 (19)	0.068 (2)	0.059 (2)	0.0185 (17)	0.0087 (17)	0.023 (2)
C13	0.047 (2)	0.058 (2)	0.045 (2)	0.0164 (17)	0.0047 (16)	0.0128 (17)
C14	0.054 (2)	0.049 (2)	0.081 (3)	0.0210 (18)	0.005 (2)	0.010 (2)
C15	0.042 (2)	0.062 (2)	0.081 (3)	0.0212 (18)	0.001 (2)	0.014 (2)
C16	0.058 (3)	0.121 (4)	0.121 (5)	0.017 (3)	0.001 (3)	0.051 (4)
C17	0.103 (5)	0.112 (4)	0.169 (7)	0.038 (4)	0.037 (4)	0.084 (5)
C18	0.138 (4)	0.143 (4)	0.133 (4)	0.039 (3)	0.052 (4)	0.006 (3)
C19	0.0434 (19)	0.059 (2)	0.057 (2)	0.0278 (16)	0.0193 (17)	0.0236 (18)
C20	0.063 (3)	0.082 (3)	0.054 (2)	0.040 (2)	0.014 (2)	0.016 (2)
C21	0.072 (3)	0.124 (4)	0.064 (3)	0.056 (3)	0.024 (2)	0.020 (3)
C22	0.071 (3)	0.155 (5)	0.069 (3)	0.058 (4)	0.038 (3)	0.055 (4)
C23	0.051 (2)	0.119 (4)	0.102 (4)	0.039 (3)	0.037 (3)	0.072 (3)
C24	0.0408 (19)	0.076 (2)	0.077 (3)	0.0314 (18)	0.0240 (19)	0.043 (2)
C25	0.099 (4)	0.065 (3)	0.099 (4)	0.030 (3)	0.028 (3)	0.007 (3)
C26	0.056 (3)	0.057 (2)	0.114 (4)	0.0227 (19)	0.035 (3)	0.028 (2)
C27	0.0333 (16)	0.0490 (18)	0.0417 (17)	0.0153 (14)	0.0126 (14)	0.0206 (15)
C28	0.0367 (17)	0.0458 (17)	0.0424 (18)	0.0133 (14)	0.0109 (14)	0.0161 (15)
C29	0.0344 (17)	0.0533 (19)	0.052 (2)	0.0083 (15)	0.0074 (15)	0.0214 (17)
C30	0.0361 (18)	0.065 (2)	0.058 (2)	0.0209 (16)	0.0189 (16)	0.0288 (19)
C31	0.0392 (18)	0.0526 (19)	0.054 (2)	0.0217 (15)	0.0199 (16)	0.0248 (17)
C32	0.055 (2)	0.052 (2)	0.050 (2)	0.0176 (17)	0.0146 (17)	0.0138 (17)
C33	0.045 (2)	0.068 (2)	0.080 (3)	−0.0019 (18)	0.008 (2)	0.027 (2)
C34	0.048 (2)	0.093 (3)	0.098 (3)	0.033 (2)	0.037 (2)	0.044 (3)
C35	0.068 (3)	0.059 (2)	0.084 (3)	0.036 (2)	0.038 (2)	0.028 (2)
C36	0.0364 (16)	0.0409 (16)	0.0453 (18)	0.0153 (13)	0.0171 (14)	0.0154 (14)
C37	0.049 (2)	0.058 (2)	0.047 (2)	0.0098 (16)	0.0198 (17)	0.0171 (17)
C38	0.044 (2)	0.060 (2)	0.054 (2)	0.0027 (16)	0.0172 (17)	0.0139 (18)
C39	0.049 (2)	0.0415 (17)	0.056 (2)	0.0137 (15)	0.0269 (17)	0.0137 (16)
C40	0.053 (2)	0.063 (2)	0.054 (2)	0.0131 (18)	0.0242 (18)	0.0263 (19)
C41	0.0428 (19)	0.067 (2)	0.046 (2)	0.0119 (17)	0.0115 (16)	0.0209 (18)
C42	0.064 (3)	0.060 (2)	0.103 (4)	0.010 (2)	0.025 (3)	0.026 (3)
C43	0.095 (4)	0.088 (3)	0.093 (4)	0.017 (3)	0.043 (3)	0.048 (3)
C44	0.100 (4)	0.084 (3)	0.171 (6)	0.046 (3)	0.092 (4)	0.042 (4)

### Geometric parameters (Å, °)

**Table:**

Ti1—O1	1.794 (2)	C17—H17C	0.9600
Ti1—O2	1.855 (2)	C18—H18A	0.9600
Ti1—Cl1	2.2933 (17)	C18—H18B	0.9600
Ti1—C1	2.355 (4)	C18—H18C	0.9600
Ti1—C5	2.363 (4)	C19—C24	1.389 (5)
Ti1—C4	2.373 (4)	C19—C20	1.395 (5)
Ti1—C3	2.384 (4)	C20—C21	1.408 (6)
Ti1—C2	2.401 (4)	C20—C25	1.500 (6)
Ti2—O2	1.784 (2)	C21—C22	1.361 (7)
Ti2—Cl3	2.2655 (13)	C21—H21	0.9300
Ti2—Cl2	2.2741 (17)	C22—C23	1.362 (7)
Ti2—C29	2.361 (4)	C22—H22	0.9300
Ti2—C28	2.367 (3)	C23—C24	1.396 (6)
Ti2—C27	2.376 (3)	C23—H23	0.9300
Ti2—C30	2.398 (4)	C24—C26	1.509 (6)
Ti2—C31	2.414 (3)	C25—H25A	0.9600
Si1—C18	1.833 (7)	C25—H25B	0.9600
Si1—C17	1.839 (6)	C25—H25C	0.9600
Si1—C16	1.854 (5)	C26—H26A	0.9600
Si1—C13	1.880 (4)	C26—H26B	0.9600
Si2—C44	1.850 (5)	C26—H26C	0.9600
Si2—C42	1.862 (5)	C27—C28	1.416 (4)
Si2—C43	1.871 (5)	C27—C31	1.431 (4)
Si2—C39	1.887 (3)	C27—C36	1.493 (4)
O1—C19	1.360 (4)	C28—C29	1.427 (4)
C1—C5	1.420 (5)	C28—C32	1.500 (5)
C1—C2	1.436 (5)	C29—C30	1.422 (5)
C1—C10	1.482 (5)	C29—C33	1.492 (5)
C2—C3	1.407 (6)	C30—C31	1.412 (5)
C2—C9	1.497 (6)	C30—C34	1.495 (5)
C3—C4	1.408 (6)	C31—C35	1.490 (5)
C3—C8	1.507 (5)	C32—H32A	0.9600
C4—C5	1.413 (5)	C32—H32B	0.9600
C4—C7	1.525 (6)	C32—H32C	0.9600
C5—C6	1.505 (6)	C33—H33A	0.9600
C6—H6A	0.9600	C33—H33B	0.9600
C6—H6B	0.9600	C33—H33C	0.9600
C6—H6C	0.9600	C34—H34A	0.9600
C7—H7A	0.9600	C34—H34B	0.9600
C7—H7B	0.9600	C34—H34C	0.9600
C7—H7C	0.9600	C35—H35A	0.9600
C8—H8A	0.9600	C35—H35B	0.9600
C8—H8B	0.9600	C35—H35C	0.9600
C8—H8C	0.9600	C36—C41	1.381 (4)
C9—H9A	0.9600	C36—C37	1.384 (5)
C9—H9B	0.9600	C37—C38	1.395 (5)
C9—H9C	0.9600	C37—H37	0.9300
C10—C11	1.386 (5)	C38—C39	1.378 (5)
C10—C15	1.405 (5)	C38—H38	0.9300
C11—C12	1.387 (5)	C39—C40	1.392 (5)
C11—H11	0.9300	C40—C41	1.397 (5)
C12—C13	1.389 (5)	C40—H40	0.9300
C12—H12	0.9300	C41—H41	0.9300
C13—C14	1.384 (5)	C42—H42A	0.9600
C14—C15	1.372 (5)	C42—H42B	0.9600
C14—H14	0.9300	C42—H42C	0.9600
C15—H15	0.9300	C43—H43A	0.9600
C16—H16A	0.9600	C43—H43B	0.9600
C16—H16B	0.9600	C43—H43C	0.9600
C16—H16C	0.9600	C44—H44A	0.9600
C17—H17A	0.9600	C44—H44B	0.9600
C17—H17B	0.9600	C44—H44C	0.9600
			
O1—Ti1—O2	105.50 (11)	C14—C15—H15	119.9
O1—Ti1—Cl1	100.53 (10)	C10—C15—H15	119.9
O2—Ti1—Cl1	101.71 (9)	Si1—C16—H16A	109.5
O1—Ti1—C1	103.29 (12)	Si1—C16—H16B	109.5
O2—Ti1—C1	97.86 (13)	H16A—C16—H16B	109.5
Cl1—Ti1—C1	143.69 (9)	Si1—C16—H16C	109.5
O1—Ti1—C5	87.20 (13)	H16A—C16—H16C	109.5
O2—Ti1—C5	132.54 (13)	H16B—C16—H16C	109.5
Cl1—Ti1—C5	121.02 (10)	Si1—C17—H17A	109.5
C1—Ti1—C5	35.03 (13)	Si1—C17—H17B	109.5
O1—Ti1—C4	107.17 (15)	H17A—C17—H17B	109.5
O2—Ti1—C4	142.97 (14)	Si1—C17—H17C	109.5
Cl1—Ti1—C4	89.04 (10)	H17A—C17—H17C	109.5
C1—Ti1—C4	57.92 (14)	H17B—C17—H17C	109.5
C5—Ti1—C4	34.71 (13)	Si1—C18—H18A	109.5
O1—Ti1—C3	141.30 (15)	Si1—C18—H18B	109.5
O2—Ti1—C3	110.16 (15)	H18A—C18—H18B	109.5
Cl1—Ti1—C3	86.69 (11)	Si1—C18—H18C	109.5
C1—Ti1—C3	57.78 (13)	H18A—C18—H18C	109.5
C5—Ti1—C3	57.58 (14)	H18B—C18—H18C	109.5
C4—Ti1—C3	34.43 (15)	O1—C19—C24	118.4 (3)
O1—Ti1—C2	138.44 (13)	O1—C19—C20	119.4 (3)
O2—Ti1—C2	86.50 (13)	C24—C19—C20	122.1 (4)
Cl1—Ti1—C2	116.07 (10)	C19—C20—C21	117.3 (4)
C1—Ti1—C2	35.15 (12)	C19—C20—C25	122.3 (4)
C5—Ti1—C2	57.90 (14)	C21—C20—C25	120.4 (4)
C4—Ti1—C2	57.31 (16)	C22—C21—C20	121.2 (5)
C3—Ti1—C2	34.19 (14)	C22—C21—H21	119.4
O2—Ti2—Cl3	103.14 (8)	C20—C21—H21	119.4
O2—Ti2—Cl2	104.50 (9)	C21—C22—C23	120.2 (4)
Cl3—Ti2—Cl2	100.73 (6)	C21—C22—H22	119.9
O2—Ti2—C29	110.69 (13)	C23—C22—H22	119.9
Cl3—Ti2—C29	88.59 (9)	C22—C23—C24	121.6 (5)
Cl2—Ti2—C29	140.43 (10)	C22—C23—H23	119.2
O2—Ti2—C28	88.37 (11)	C24—C23—H23	119.2
Cl3—Ti2—C28	120.73 (9)	C19—C24—C23	117.6 (4)
Cl2—Ti2—C28	132.64 (8)	C19—C24—C26	120.6 (3)
C29—Ti2—C28	35.13 (11)	C23—C24—C26	121.8 (4)
O2—Ti2—C27	101.71 (11)	C20—C25—H25A	109.5
Cl3—Ti2—C27	143.82 (9)	C20—C25—H25B	109.5
Cl2—Ti2—C27	98.01 (9)	H25A—C25—H25B	109.5
C29—Ti2—C27	58.00 (11)	C20—C25—H25C	109.5
C28—Ti2—C27	34.73 (11)	H25A—C25—H25C	109.5
O2—Ti2—C30	144.44 (12)	H25B—C25—H25C	109.5
Cl3—Ti2—C30	87.46 (9)	C24—C26—H26A	109.5
Cl2—Ti2—C30	106.69 (10)	C24—C26—H26B	109.5
C29—Ti2—C30	34.75 (13)	H26A—C26—H26B	109.5
C28—Ti2—C30	57.77 (12)	C24—C26—H26C	109.5
C27—Ti2—C30	57.46 (11)	H26A—C26—H26C	109.5
O2—Ti2—C31	136.29 (11)	H26B—C26—H26C	109.5
Cl3—Ti2—C31	117.28 (9)	C28—C27—C31	108.2 (3)
Cl2—Ti2—C31	84.51 (9)	C28—C27—C36	126.1 (3)
C29—Ti2—C31	57.56 (12)	C31—C27—C36	125.1 (3)
C28—Ti2—C31	57.68 (12)	C28—C27—Ti2	72.31 (17)
C27—Ti2—C31	34.76 (11)	C31—C27—Ti2	74.09 (18)
C30—Ti2—C31	34.11 (11)	C36—C27—Ti2	126.2 (2)
C18—Si1—C17	109.7 (4)	C27—C28—C29	107.8 (3)
C18—Si1—C16	109.5 (3)	C27—C28—C32	126.7 (3)
C17—Si1—C16	109.2 (3)	C29—C28—C32	125.4 (3)
C18—Si1—C13	108.6 (3)	C27—C28—Ti2	72.96 (18)
C17—Si1—C13	108.8 (2)	C29—C28—Ti2	72.18 (19)
C16—Si1—C13	110.9 (2)	C32—C28—Ti2	123.3 (2)
C44—Si2—C42	108.6 (3)	C30—C29—C28	107.8 (3)
C44—Si2—C43	111.7 (3)	C30—C29—C33	126.1 (3)
C42—Si2—C43	109.3 (2)	C28—C29—C33	125.9 (4)
C44—Si2—C39	109.29 (19)	C30—C29—Ti2	74.1 (2)
C42—Si2—C39	109.38 (19)	C28—C29—Ti2	72.69 (19)
C43—Si2—C39	108.5 (2)	C33—C29—Ti2	122.1 (3)
C19—O1—Ti1	174.5 (2)	C31—C30—C29	108.5 (3)
Ti2—O2—Ti1	157.02 (14)	C31—C30—C34	125.3 (4)
C5—C1—C2	107.7 (3)	C29—C30—C34	126.1 (3)
C5—C1—C10	126.2 (3)	C31—C30—Ti2	73.58 (19)
C2—C1—C10	125.9 (4)	C29—C30—Ti2	71.2 (2)
C5—C1—Ti1	72.8 (2)	C34—C30—Ti2	124.2 (3)
C2—C1—Ti1	74.2 (2)	C30—C31—C27	107.6 (3)
C10—C1—Ti1	123.1 (2)	C30—C31—C35	125.8 (3)
C3—C2—C1	107.3 (4)	C27—C31—C35	126.1 (3)
C3—C2—C9	125.8 (4)	C30—C31—Ti2	72.31 (19)
C1—C2—C9	126.7 (4)	C27—C31—Ti2	71.15 (18)
C3—C2—Ti1	72.3 (2)	C35—C31—Ti2	128.0 (3)
C1—C2—Ti1	70.7 (2)	C28—C32—H32A	109.5
C9—C2—Ti1	126.0 (3)	C28—C32—H32B	109.5
C2—C3—C4	108.8 (3)	H32A—C32—H32B	109.5
C2—C3—C8	124.7 (5)	C28—C32—H32C	109.5
C4—C3—C8	126.4 (4)	H32A—C32—H32C	109.5
C2—C3—Ti1	73.5 (2)	H32B—C32—H32C	109.5
C4—C3—Ti1	72.4 (2)	C29—C33—H33A	109.5
C8—C3—Ti1	123.2 (3)	C29—C33—H33B	109.5
C3—C4—C5	108.3 (4)	H33A—C33—H33B	109.5
C3—C4—C7	126.1 (4)	C29—C33—H33C	109.5
C5—C4—C7	125.5 (4)	H33A—C33—H33C	109.5
C3—C4—Ti1	73.2 (2)	H33B—C33—H33C	109.5
C5—C4—Ti1	72.3 (2)	C30—C34—H34A	109.5
C7—C4—Ti1	123.1 (3)	C30—C34—H34B	109.5
C4—C5—C1	107.8 (4)	H34A—C34—H34B	109.5
C4—C5—C6	126.2 (4)	C30—C34—H34C	109.5
C1—C5—C6	125.9 (3)	H34A—C34—H34C	109.5
C4—C5—Ti1	73.0 (2)	H34B—C34—H34C	109.5
C1—C5—Ti1	72.2 (2)	C31—C35—H35A	109.5
C6—C5—Ti1	121.5 (3)	C31—C35—H35B	109.5
C5—C6—H6A	109.5	H35A—C35—H35B	109.5
C5—C6—H6B	109.5	C31—C35—H35C	109.5
H6A—C6—H6B	109.5	H35A—C35—H35C	109.5
C5—C6—H6C	109.5	H35B—C35—H35C	109.5
H6A—C6—H6C	109.5	C41—C36—C37	118.2 (3)
H6B—C6—H6C	109.5	C41—C36—C27	118.7 (3)
C4—C7—H7A	109.5	C37—C36—C27	123.1 (3)
C4—C7—H7B	109.5	C36—C37—C38	120.1 (3)
H7A—C7—H7B	109.5	C36—C37—H37	119.9
C4—C7—H7C	109.5	C38—C37—H37	119.9
H7A—C7—H7C	109.5	C39—C38—C37	122.7 (4)
H7B—C7—H7C	109.5	C39—C38—H38	118.6
C3—C8—H8A	109.5	C37—C38—H38	118.6
C3—C8—H8B	109.5	C38—C39—C40	116.5 (3)
H8A—C8—H8B	109.5	C38—C39—Si2	122.1 (3)
C3—C8—H8C	109.5	C40—C39—Si2	121.4 (3)
H8A—C8—H8C	109.5	C39—C40—C41	121.5 (3)
H8B—C8—H8C	109.5	C39—C40—H40	119.3
C2—C9—H9A	109.5	C41—C40—H40	119.3
C2—C9—H9B	109.5	C36—C41—C40	121.0 (3)
H9A—C9—H9B	109.5	C36—C41—H41	119.5
C2—C9—H9C	109.5	C40—C41—H41	119.5
H9A—C9—H9C	109.5	Si2—C42—H42A	109.5
H9B—C9—H9C	109.5	Si2—C42—H42B	109.5
C11—C10—C15	117.6 (3)	H42A—C42—H42B	109.5
C11—C10—C1	120.3 (3)	Si2—C42—H42C	109.5
C15—C10—C1	122.0 (3)	H42A—C42—H42C	109.5
C10—C11—C12	121.2 (3)	H42B—C42—H42C	109.5
C10—C11—H11	119.4	Si2—C43—H43A	109.5
C12—C11—H11	119.4	Si2—C43—H43B	109.5
C11—C12—C13	121.4 (3)	H43A—C43—H43B	109.5
C11—C12—H12	119.3	Si2—C43—H43C	109.5
C13—C12—H12	119.3	H43A—C43—H43C	109.5
C14—C13—C12	116.8 (3)	H43B—C43—H43C	109.5
C14—C13—Si1	120.1 (3)	Si2—C44—H44A	109.5
C12—C13—Si1	123.1 (3)	Si2—C44—H44B	109.5
C15—C14—C13	122.8 (4)	H44A—C44—H44B	109.5
C15—C14—H14	118.6	Si2—C44—H44C	109.5
C13—C14—H14	118.6	H44A—C44—H44C	109.5
C14—C15—C10	120.2 (4)	H44B—C44—H44C	109.5
